# White LED Light Radiation Promotes Growth and Phytochemical Accumulation in Moringa Seedlings as Promising Functional Microgreen

**DOI:** 10.3390/foods15213960

**Published:** 2026-11-04

**Authors:** Huan Chen, Lijing Liu, Chunyan Zhao, Xiaoli Zhang, Xi Qi, Jiming Long, Jiaqi Shen, Xinrui Kang, Xiaoping Fu, Yang Tian, Tong Zhu

**Affiliations:** 1 College of Food Science and Technology, Yunnan Agricultural University, Kunming 650201, China; 15198762463@163.com (H.C.); lilyliutt@163.com (L.L.); 18725236145@163.com (X.Z.); remember5forget5@163.com (X.Q.); xihe0718@163.com (X.K.); tianyang1208@163.com (Y.T.); 2 College of Tea Science, Yunnan Agricultural University, Kunming 650201, China; zcy19940726@126.com; 3 Yunnan Institute of Tropical Crops, Jinghong 666100, China; ljm110112@163.com; 4 Institute of Dryland Crops, Guizhou Academy of Agricultural Sciences, Guiyang 550006, China; gzsjq1990@163.com

**Keywords:** white LED light, moringa seedlings, growth promotion, phytochemical enrichment, phenylpropanoid pathway, functional microgreen

## Abstract

This study investigated the effects of white light-emitting diode (LED) radiation on the growth and phytochemical accumulation of moringa seedlings. Specifically, we identified the optimal light radiation conditions (intensity and photoperiod) for growth, fresh weight accumulation, and physiological performance of moringa seedlings. We further elucidated the changes in the functional properties of and mechanisms underlying phytochemical accumulation in moringa seedlings under light radiation. The results showed that under the optimal light radiation conditions (5 μmol/m^2^·s and 8 h/d), the fresh weight, hypocotyl length, and respiratory intensity of moringa seedlings increased significantly, while electrolyte leakage decreased significantly. Light radiation markedly enhanced the contents of total phenolics, total flavonoids, and specific phytochemicals (quercetin 3-O-(6-O-malonyl-β-D-glucoside), phloretin, 1-O-vanilloyl-β-D-glucose, 4-coumaric acid, and 3-coumaric acid); enhanced bioactivity-related functional properties, including 1,1-diphenyl-2-picrylhydrazyl (DPPH) free radical scavenging activity, superoxide radical absorbance capacity (SORAC); and enhanced the inhibitory activities of α-glucosidase, pancreatic lipase, and angiotensin-converting enzyme during the growth of moringa seedlings. The expression of genes (*Mo4CL*, *MoF3H*, *MoLAR*) and the activities of enzymes (PAL, C4H, 4CL) involved in the phenylpropanoid pathway were also markedly upregulated. Overall, white LED light radiation (5 μmol/m^2^·s and 8 h/d) can be used as an effective approach to promote the growth of and phytochemical accumulation in moringa seedlings, supporting their potential as a functional microgreen.

## 1. Introduction

Moringa (*Moringa oleifera* Lam.), belonging to the Moringaceae family, originates from India [1]. Due to its multiple uses, such as a source of food and medicine, a coagulant for water purification, a biostimulant, and a biofuel source, moringa is widely cultivated in tropical and subtropical regions including India, Pakistan, Central and South America, Cambodia, the Philippines, and China, among others [2]. Notably, almost all parts (roots, stems, leaves, flowers, fruits and seeds) of moringa are edible, and moringa has a long history of consumption [3]. Studies have shown that moringa is rich in various kinds of nutrients (dietary fiber, polyunsaturated fatty acids, essential amino acids, vitamins, and minerals) and bioactive compounds (alkaloids, saponins, steroids, tannins, polyphenols, flavonoids, anthocyanins. and glucosinolates) [4,5]. Various beneficial effects of moringa have been reported, including antioxidant, anti-inflammatory, cardioprotective, antidiabetic, antimicrobial, neuroprotective, anticancer, and gut microbiota regulation effects [6].

Microgreens, which are tender immature greens, are germinated seedlings with fully developed and non-senescent cotyledons and emerging or developing true leaves [7]. Due to their high nutrient content, microgreens are increasingly popular in modern diets, and their advantages include a short production cycle (7 to 21 days), year-round production in plant factories (without pesticides and occupying arable land), and enrichment in health-promoting phytochemicals [8,9]. More importantly, during germination, endogenous enzymes in seeds are activated, and their activities increase significantly, resulting to the breakdown (catabolism) of stored macronutrients (polysaccharides, fats and proteins) into digestible and bioaccessible compounds (soluble sugars, fatty acids and amino acids), along with a reduction in antinutrients [10,11]. Moreover, the increase in catabolic simple molecules (glucose and free amino acids) promotes the accumulation of secondary metabolites, as glucose and free amino acids serve as precursors for phenolic and flavonoid synthesis through pathways such as the oxidative pentose phosphate, glycolysis, and shikimate pathways and the Krebs cycle [12]. Light radiation (wavelength, intensity and photoperiod) plays an integral role in virtually all aspects of plant life, affecting plant development, physiology and survival [13]. Studies have applied light radiation in microgreen cultivation, which have shown that it has enhanced growth, biomass accumulation, and the contents of nutrients and phytochemicals in microgreens such as chia, arugula, cabbage, basil, carrot and radish [14,15,16].

Although moringa seedlings have a long history of dietary consumption and are favored by consumers, research on the regulation of their growth, physiology, and phytochemical accumulation remains limited. Currently, only a few studies have investigated the optimization of the germination conditions for moringa sprouts or have analyzed the nutrient composition, bioactive compounds, and antioxidant activity of moringa sprouts or seedlings [17,18,19]. In most of these studies, lighting conditions were not considered during the cultivation of moringa microgreens. Moreover, nutritional and bioactive compound analyses were generally limited to broad categories of basic nutrients (carbohydrates, proteins and lipids) and bioactive compounds (total polyphenols and flavonoids). Furthermore, the functional analysis of moringa microgreens has mainly focused on antioxidant activity, including 1,1-diphenyl-2-picrylhydrazyl (DPPH) free-radical scavenging activity and the ferric reducing antioxidant power (FRAP) assay. Thus, the effects of light radiation on the growth of and physiological changes in moringa microgreens during cultivation remain unclear, and further investigation is needed to elucidate changes in specific phytochemicals and functional properties beyond antioxidant activity. Based on the above research gaps, the aims of this study were as follows: to explore the effects of light intensity and photoperiod on growth and physiological parameters during moringa seedling cultivation; to analyze the changes in the phytochemicals and functional properties during moringa seedling cultivation and construct a correlation network between functional properties and phytochemicals to identify key phytochemicals associated with these properties; and, finally, to clarify the mechanisms through which key phytochemicals accumulate by examining the enzyme activities and gene expression in the relevant biosynthetic pathways. Theoretically, the results broaden our understanding of the mechanisms through which phytochemicals accumulate during moringa seedling cultivation and the biological roles of light radiation in regulating the growth and phytochemical accumulation of microgreens. Practically, the results provide guidance for the large-scale industrial production of moringa seedlings as a promising functional microgreen.

## 2. Materials and Methods

### 2.1. Materials

Moringa seeds (*Moringa oleifera* Lam.) of the cultivar PKM-1 harvested in October 2023 were provided by the Yunnan Institute of Tropical Crops and stored at −18 °C. White light-emitting diode (LED) lamps (30/T8 10 W, Hongtaiyang Co., Zhongshan, China) were used to simulate visible light, and the spectral energy distribution is shown in Supplementary Figure S1. An O_2_^−^ assay kit (cat. No. A052-1-1, Nanjing Jiancheng Biotech Co., Nanjing, China) and an α-glucosidase inhibition activity assay kit (cat. no. BC6380, Solarbio Co., Beijing, China) were used for the respective analyses.

### 2.2. Moringa Seedling Cultivation

**Figure 1:**
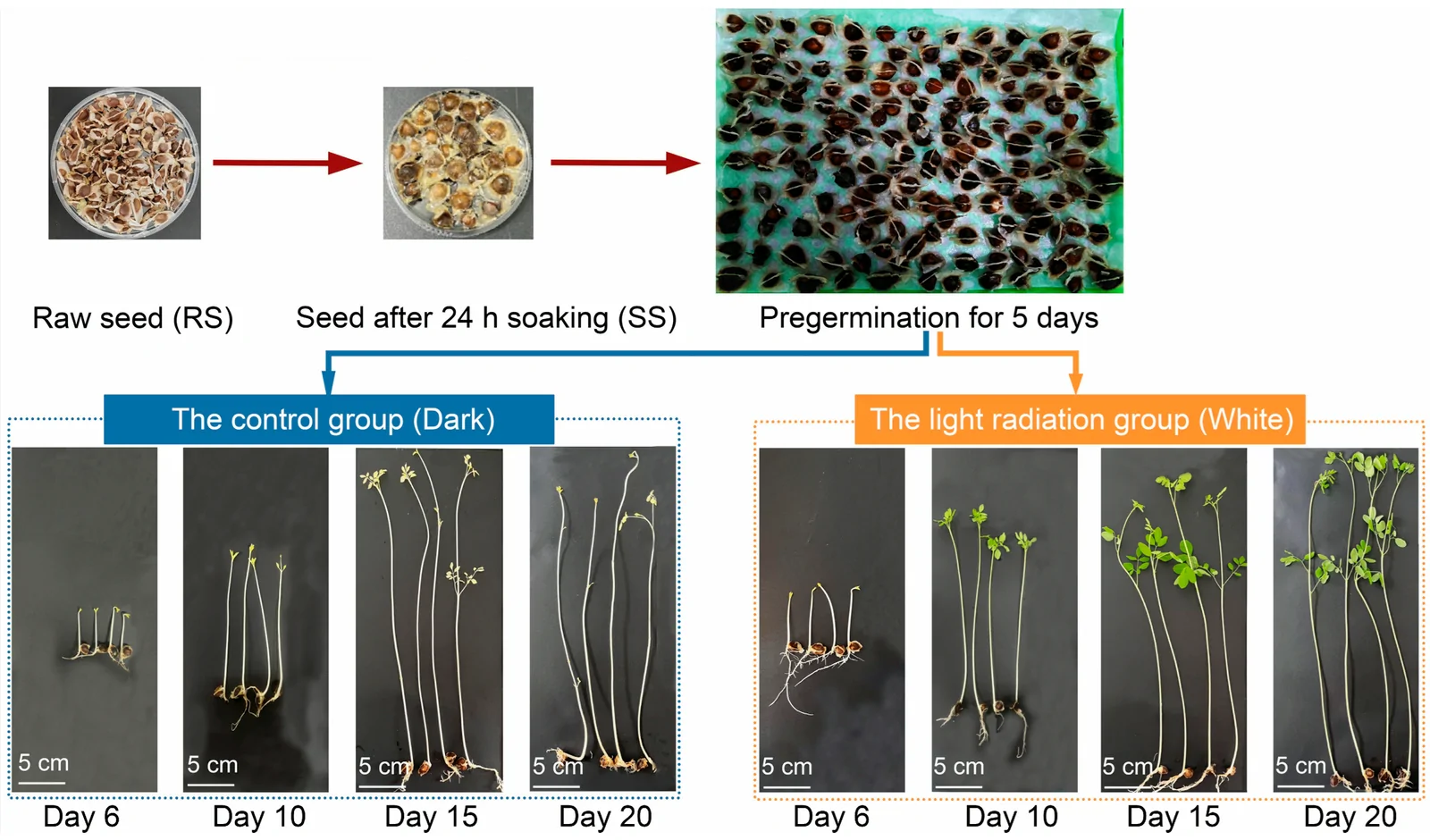
Experimental protocol. Moringa samples were collected at different growth stages, including raw seeds (RSs), seeds after 24 h of soaking (SSs), and seedlings on days 6, 10, 15, and 20 of growth for subsequent analyses.

Moringa seeds of uniform size and free from disease and mechanical damage were selected and surface-sterilized with 5% (*v*/*v*) sodium hypochlorite for 10 min, followed by rinsing three times with deionized water. Subsequently, moringa seeds were soaked in deionized water at (30 ± 1) °C for 24 h and then transferred to multihole plastic dishes for 5 days of pregermination at (25 ± 1) °C and (55 ± 5)% relative humidity (RH). After 5 days of pregermination, uniformly germinated moringa seeds (with radicle lengths of 1–2 cm) were selected and cultivated in growth chambers (PCTHI-250T, STIK Co., Shanghai, China) at (25 ± 1) °C and (55 ± 5)% RH for subsequent experiments, which were divided into three parts. First, germinated moringa seeds were exposed to different LED intensities (0, 5, 15, 30, 60, 120 and 360 μmol/m^2^·s) in an 8 h/day photoperiod during seedling growth. Second, germinated moringa seeds were exposed to different photoperiods (0, 2, 4, 8, 12 and 24 h/d) at a fixed light intensity of (5 μmol/m^2^·s) during seedling growth. Third, germinated moringa seeds were exposed to a light intensity of 5 μmol/m^2^·s with an 8 h/d photoperiod during seedling growth. In all experiments, seedlings cultivated in darkness served as the control. Each group contained 360 uniformly pregerminated moringa sprouts, which were divided into three subgroups (120 moringa sprouts per subgroup) as replicates. The entire cultivation process is illustrated in Figure 1. Raw seeds (RSs), seeds after 24 h of soaking (SSs), and moringa seedlings on days 6, 10, 15 and 20 of growth were collected, frozen in liquid nitrogen and stored at −80 °C until subsequent analyses.

### 2.3. Determination of Growth and Physiological Parameters

An electronic balance (precision of ±0.001 g, LE203E/02, Mettler Toledo Co., Shanghai, China) and Vernier calipers (precision of ±0.02 mm, 202160-2, Vogel Co., Berlin, Germany) were used to measure the fresh weight and hypocotyl length of moringa seedlings, respectively. The results are expressed as the mean values obtained from 30 moringa seedlings across three replicates. Respiratory intensity and electrolyte leakage were measured according to the method of Yin et al. [20] and Dionisio et al. [21], respectively, and are expressed in mg CO_2_/(g·h) and %, respectively.

### 2.4. Assessment of Antioxidants and Functional-Related Properties

The extraction and measurement of total phenolics, total flavonoids, 1,1-diphenyl-2-picrylhydrazyl (DPPH) free-radical scavenging activity and superoxide radical absorbance capacity (SORAC) were performed according to our previously reported method [22], with the results expressed as gallic acid equivalents (mg GAE/g dry weight (DW)), rutin equivalents (mg rutin/g DW), Trolox equivalents (mM Trolox/g DW) and U/g fresh weight (FW), respectively.

A 0.1 g sample of freeze-dried moringa seedling powder was homogenized with 5 mL of an extraction solution (acetone: ethanol: water; 2:2:1, *v*/*v*) containing 0.1% (*v*/*v*) acetic acid and then extracted in a shaking incubator at 200 rpm and room temperature for 12 h. Subsequently, the supernatant was collected by centrifugation at 12,000× *g* at 4 °C for 15 min, followed by drying at 40 °C and redissolution in 1 mL of dimethyl sulfoxide for the subsequent measurements.

α-Glucosidase inhibition was determined using an α-glucosidase inhibition activity assay kit (cat. no. BC6380, Solarbio Co., Beijing, China), and the results are expressed as a percentage (%).

Pancreatic lipase inhibition was measured according to the method of Gilham et al. [23], using *p*-nitrophenyl laurate (*p*-NPL) as the substrate, with some modifications. A 30 μL aliquot of the sample extract was mixed with 50 μL of Tris-HCl buffer (0.1 mol/L, pH 8.2), 80 μL of 0.1 mol/L Tris-HCl buffer (pH 8.2) containing 0.8 mg/mL *p*-NPL, and 60 μL of 0.1 mol/L Tris-HCl buffer (pH 8.2) containing 100 mg/mL pancreatic lipase in a 96 well-microplate. The reaction was then carried out at 37 °C for 20 min, and the absorbance at 405 nm was recorded. The percentage pancreatic lipase inhibition was calculated as follows:

$$\mathrm{Pancreatic}\;\mathrm{lipase}\;\mathrm{inhibition}\;(\%)=\left(1-\frac{A3-A4}{A1-A2}\right)\times 100$$

where A1 (control group) represents the absorbance of the reaction between pancreatic lipase and the substrate; A2 (blank control group) represents the absorbance of the substrate without pancreatic lipase; A3 (sample group) represents the absorbance of the sample mixed with pancreatic lipase and the substrate; A4 (sample blank group) represents the absorbance of the sample mixed with the substrate without pancreatic lipase.

The inhibition rate of angiotensin-converting enzyme (ACE) was determined according to the method of Ana et al. [24], and the results are expressed as a percentage (%).

### 2.5. Phytochemical Identification by UHPLC-MS/MS

Raw moringa seeds (RSs) and seedlings in the control group on day 6 (D6) and day 20 (D20) of growth and moringa seedlings in the light radiation group on day 20 (L20) of growth were selected for phytochemical identification analysis. Specifically, 50 μg of lyophilized sample powder was mixed with 800 μL of 70% MeOH (*v*/*v*) and 20 μL of a mixed internal standard solution (0.3 mg/mL of d3-Leucine, 13C9-phenylalanine, d5-tryptophan, and 13C3-progesterone). The mixture was then vortexed for 5 min, followed by sonication at 50 Hz and 4 °C for 30 min, and subsequent incubation for 1 h at −20 °C. After centrifugation at 14,000× *g* for 15 min at 4 °C, a 600 μL aliquot of the resulting supernatant was collected and passed through a 0.22 μm organic membrane filter. Instrument repeatability and stability were assessed using a pooled quality control (QC) sample, which was prepared by combining 20 μL of the filtered solution from each individual sample. The filtered samples and pooled QC sample were analyzed using an ultra-performance liquid chromatography (UPLC) system (2777C, Waters Co., Milford, MA, USA) equipped with a Hypersil GOLD aQ C18 column (PN 25302-102130, 2.1 mm × 100 mm, 1.9 μm, Thermo Fisher Scientific, Waltham, MA, USA) and coupled to a Q Exactive HF high-resolution mass spectrometer (Thermo Fisher Scientific, Waltham, MA, USA) equipped with an electrospray ionization source. The mobile phase consisted of 0.1% (*v*/*v*) formic acid in ultrapure water (phase A) and 0.1% (*v*/*v*) formic acid in acetonitrile (phase B), and a linear gradient elution was used as follows: 0–2.0 min, B: 5%; 2.0–22.0 min, B: 5–95%; 22.0–27.0 min, B: 95%; 27.1–30.0 min, B: 95%. Chromatographic separation was performed at a flow rate of 0.3 mL/min, with the column temperature maintained at 40 °C, and an injection volume of 5 μL. The mass-to-charge ratio (*m*/*z*) acquisition range was 100–1500, and phytochemical ion peak extraction and compound identification were performed using Compound Discoverer^TM^ software (version 3.3, Thermo Fisher Scientific, Waltham, MA, USA). MS/MS spectral libraries, including the BGI Metabolome Database (BMDB), mzCloud, and ChemSpider, were used to identify phytochemicals.

### 2.6. Measurement of Enzyme Activities and Gene Expression in Phenylpropanoid Biosynthesis Pathway

The activities of enzymes including PAL (EC 4.3.1.5), C4H (EC 1.14.13.11) and 4CL (EC 6.2.1.12) were determined using previously reported extraction and assay methods for PAL [25], C4H [26] and 4CL [27]. Specifically, one unit of PAL activity was defined as an increase of 0.01 in absorbance at 290 nm per hour; one unit of C4H activity was defined as an increase of 0.01 in absorbance at 340 nm per min; and one unit of 4CL activity was defined as an increase of 0.01 in absorbance at 333 nm per min. Enzyme activities were determined through three independent measurements, and the results are expressed in U/mg protein. The protein content of the crude enzyme extracts was determined using the Bradford method [28].

The expression of the genes involved in the phenylpropanoid biosynthesis pathway (*Mo4CL*, *MoCHS*, *MoCHI*, *MoF3H*, *MoDFR* and *MoLAR*) was further analyzed. The methods and kits used for RNA extraction, purification, reverse-transcription and gene expression analysis followed those described in a previous report [28]. The quantity and quality of the extracted RNA were assessed using a spectrophotometer (Nanodrop 2000, Thermo Scientific Inc., Wilmington, DE, USA) and 0.8% (*w*/*v*) agarose gel electrophoresis, respectively. Following reverse transcription, a StepOnePlus PCR system (Applied Biosystems Inc., Foster City, CA, USA) was used for gene expression analysis. The primers used are listed in Supplementary Table S4, with *MoRPL1* and *MoACP2* used as internal control genes, and the expression levels of the target genes were determined in triplicate.

### 2.7. Bioinformatics and Statistical Analyses

Results are presented as mean ± standard deviation (SD). Statistical analyses were conducted using SPSS software (version 26.0, SPSS Inc., Chicago, IL, USA), and the Shapiro–Wilk test was used to assess the normality of the data. For normally distributed data, statistical analyses were performed using an independent two-tailed *t*-test (for comparisons between two groups) or one-way analysis of variance (ANOVA) followed by Duncan’s multiple range test (for comparisons among multiple groups). For non-normally distributed data, statistical analyses were performed using the Wilcoxon test (for comparisons between two groups), with the Benjamini–Hochberg correction. A *p*-value < 0.05 was considered statistically significant. Graphs were generated using OriginPro 2025 (version 10.2.0.188 OriginLab Co., Northampton, MA, USA). After matching against the MS/MS spectral libraries, a data matrix containing metabolite peak areas and identification information was used for subsequent analyses using the Dr. Tom platform (https://biosys.bgi.com accessed on 10 June 2025), including data preprocessing, data quality control, metabolite functional annotation, screening of significantly differential metabolites, and Kyoto Encyclopedia of Genes and Genomes (KEGG) pathway enrichment analysis. Among these analyses, orthogonal partial least squares–discriminant analysis (OPLS-DA) was used to screen for differential metabolites, with the selection criteria being a variable importance in projection (VIP) value > 1 and a fold change (FC) > 1.2 or <0.83, together with a Benjamini–Hochberg-adjusted *p*-value < 0.05. Molecular ecological network (MEN) analysis was conducted to investigate the correlations between phytochemicals and functional properties using the iNAP online platform (https://inap.denglab.org.cn/root/login?redirect=%2F accessed on 20 July 2025) [29]. Furthermore, the Sparse Correlations for Compositional Data (SparCC) algorithm was used for correlation analysis, and significant correlations (*p* < 0.05) with correlation coefficients > 0.7 were used to construct a network matrix, which was subsequently visualized using Cytoscape (version 3.9.1).

## 3. Results

### 3.1. Changes in Growth and Physiological Parameters of Moringa Seedlings

**Figure 2:**
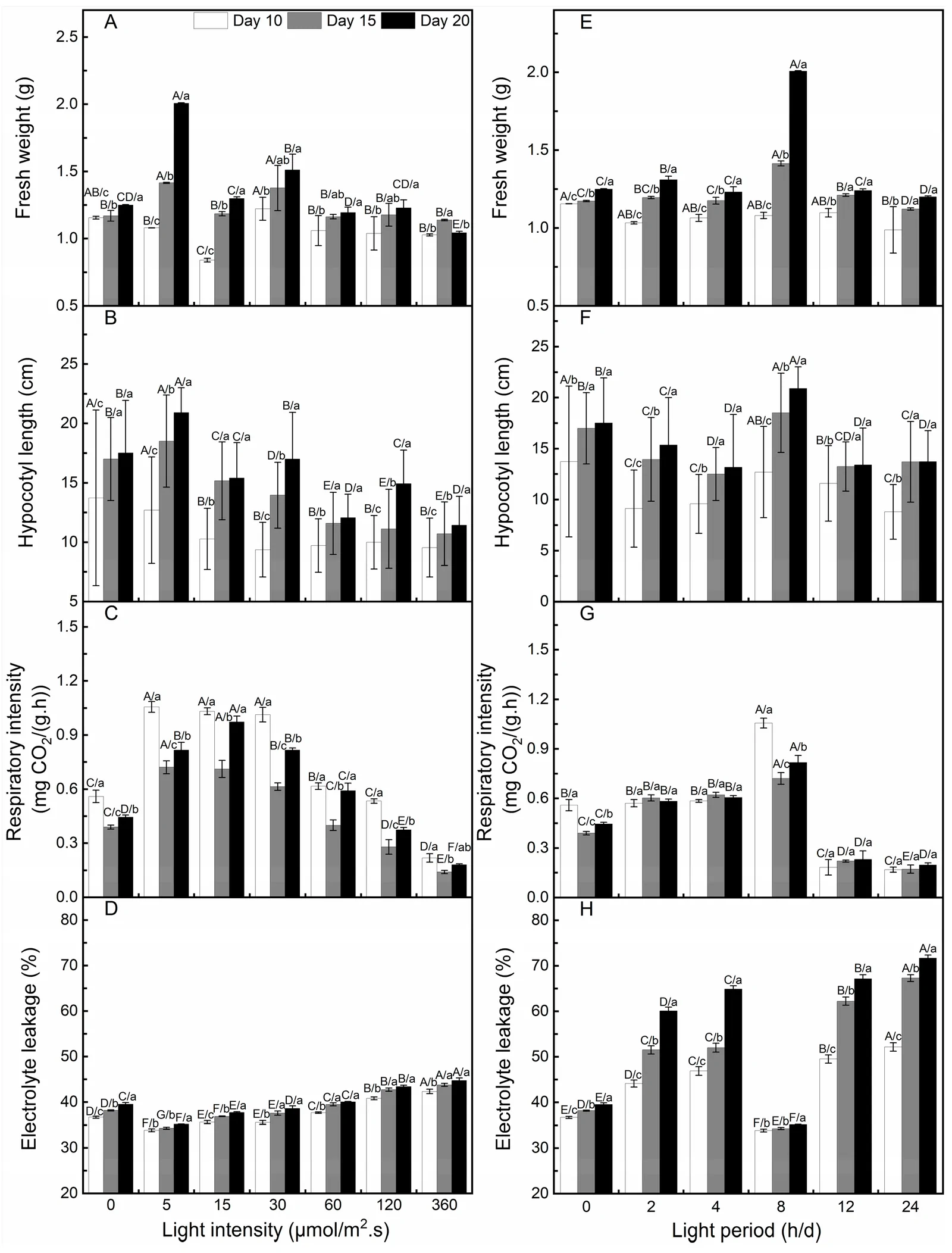
Changes in growth and physiological parameters of moringa seedlings under different light treatments. (**A**–**D**) Moringa seedlings cultivated under different light intensities at a fixed photoperiod; (**E**–**H**) moringa seedlings cultivated under different photoperiods at a fixed light intensity. Statistical analyses were performed using Duncan’s multiple range test. Different uppercase letters indicate significant differences (*p* < 0.05) among treatment groups on the same sampling day, whereas different lowercase letters indicate significant differences (*p* < 0.05) among sampling days within the same group.

Morphological changes in moringa seedlings are shown in Supplementary Figure S2. Figure 2A–D shows the growth and physiological parameters of moringa seedlings treated with different light intensities. Fresh weight increased significantly during growth, except in the group exposed to a light intensity of 360 μmol/m^2^·s. On day 20, the highest fresh weight (2.01 g) was observed in the group exposed to 5 μmol/m^2^·s, whereas the group treated with 360 μmol/m^2^·s exhibited the lowest value (1.04 g). Hypocotyl length increased significantly during seedling growth, and, on day 20, the group treated with 5 μmol/m^2^·s exhibited the longest hypocotyls (20.92 cm), while the group treated with 360 μmol/m^2^·s exhibited the shortest hypocotyls (11.43 cm). Respiratory intensity exhibited a declining trend, and the respiratory intensity of seedlings exposed to 5, 15 and 30 μmol/m^2^·s was significantly higher than that of the control during growth. However, the group treated with 360 μmol/m^2^·s exhibited the lowest respiratory intensity. Electrolyte leakage increased during moringa seedling growth. Among the treatment groups, the group exposed to 5 μmol/m^2^·s exhibited the lowest value (33.88, 34.30 and 35.18%, respectively), whereas the group exposed to 360 μmol/m^2^·s exhibited the highest values (42.34, 43.79 and 44.74%, respectively).

Based on these results, exposure to a light intensity of 5 μmol/m^2^·s significantly promoted fresh weight accumulation and hypocotyl growth while reducing electrolyte leakage. Therefore, the light intensity was fixed at 5 μmol/m^2^·s, and the changes in the growth and physiological parameters of moringa seedlings treated with different photoperiods were further investigated (Figure 2E–H), with the morphological changes shown in Supplementary Figure S3. Fresh weight, hypocotyl length, and electrolyte leakage generally increased during the growth of moringa seedlings exposed to different photoperiods. Specifically, on day 20, the group treated with an 8 h/d photoperiod exhibited the highest fresh weight (2.01 g) and longest hypocotyls (20.92 cm), as well as the lowest electrolyte leakage (35.18%). In contrast, on day 20, the group treated with a 24 h/d photoperiod exhibited the lowest fresh weight (1.20 g) and the highest electrolyte leakage (71.68%). During moringa seedling growth, no significant differences in respiratory intensity were observed among the groups treated with photoperiods of 2, 4 and 12 h/d. Furthermore, the group treated with an 8 h/d photoperiod exhibited the highest respiratory intensity, whereas the group treated with a 24 h/d photoperiod exhibited the lowest respiratory intensity throughout the seedling growth period.

### 3.2. Antioxidants and Functional Properties Upregulated During Moringa Seedling Growth

**Figure 3:**
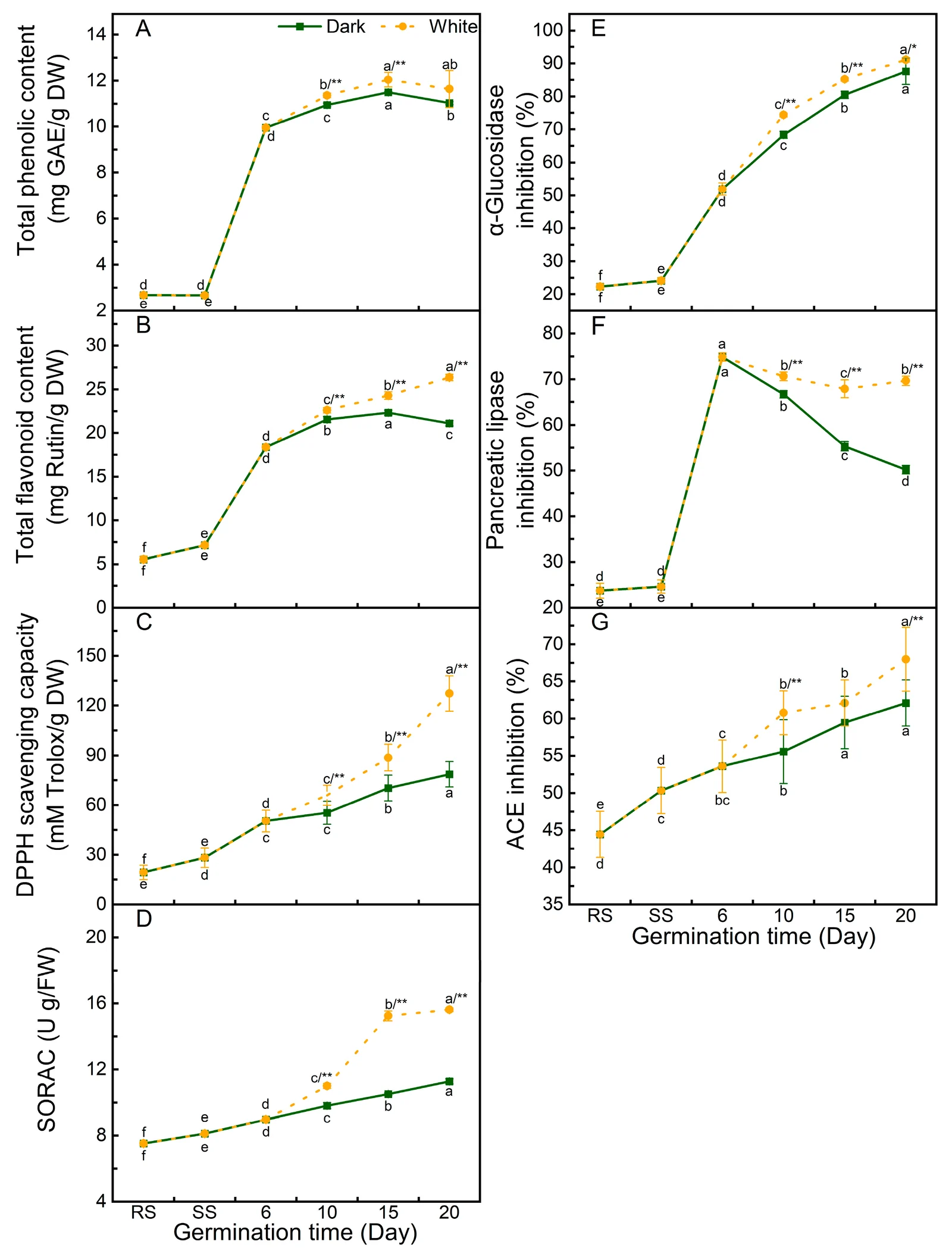
Changes in antioxidant contents, antioxidant capacities, and functional properties of moringa seedlings during growth. (**A**) Total phenolics; (**B**) total flavonoids; (**C**) DPPH scavenging capacity; (**D**) SORAC; (**E**) α-glucosidase inhibition; (**F**) pancreatic lipase inhibition; (**G**) ACE inhibition. Statistical analysis analyses were performed using an independent two-tailed *t*-test (two-group comparisons) and Duncan’s multiple range test (comparisons among multiple groups). Different lowercase letters indicate significant differences (*p* < 0.05) among sampling days within the same group, whereas asterisks indicate significant differences (* *p* < 0.05, ** *p* < 0.01) between the control and light-treated group on the same sampling day.

Overall, antioxidant contents and functional properties generally showed an increasing trend (Figure 3). The highest total phenolic contents were observed on day 15, reaching 11.49 and 12.04 mg GAE/g DW in the dark control and white-light-treated groups, respectively. From days 10 to 15, the total phenolic content in the white light treatment group was notably higher than that in the control. From days 10 to 20, the total flavonoid content, DPPH scavenging capacity, SORAC, α-glucosidase inhibition, and pancreatic lipase inhibition in the light-treated group were noticeably higher than those in the control (Figure 3B–F). Moreover, in both the control and white-light-treated groups, the highest values of DPPH scavenging capacity, SORAC, and α-glucosidase inhibition were observed on day 20, reaching 78.63 mmol Trolox/g DW, 11.28 U/L FW, and 87.65%, respectively, in the control group, and 127.32 mmol Trolox/g DW, 15.63 U/L FW, and 91.12%, respectively, in the light-treated group. Regarding total flavonoids, the highest content in the light-treated group was observed on day 20 (26.37 mg rutin/g DW), whereas the highest content in the control group was observed on day 15 (22.32 mg rutin/g). However, the highest pancreatic lipase inhibition rate in both groups was observed on day 6, reaching 74.86% in both control and light-treated groups. Compared with the control, ACE inhibition was significantly higher in the light-treated group on days 10 and 20 (Figure 3G), with the highest ACE inhibition observed on day 20 (62.09% in the control group and 67.97% in the light-treated group).

### 3.3. Phytochemical Accumulation Upregulated During Moringa Seedling Growth

**Figure 4:**
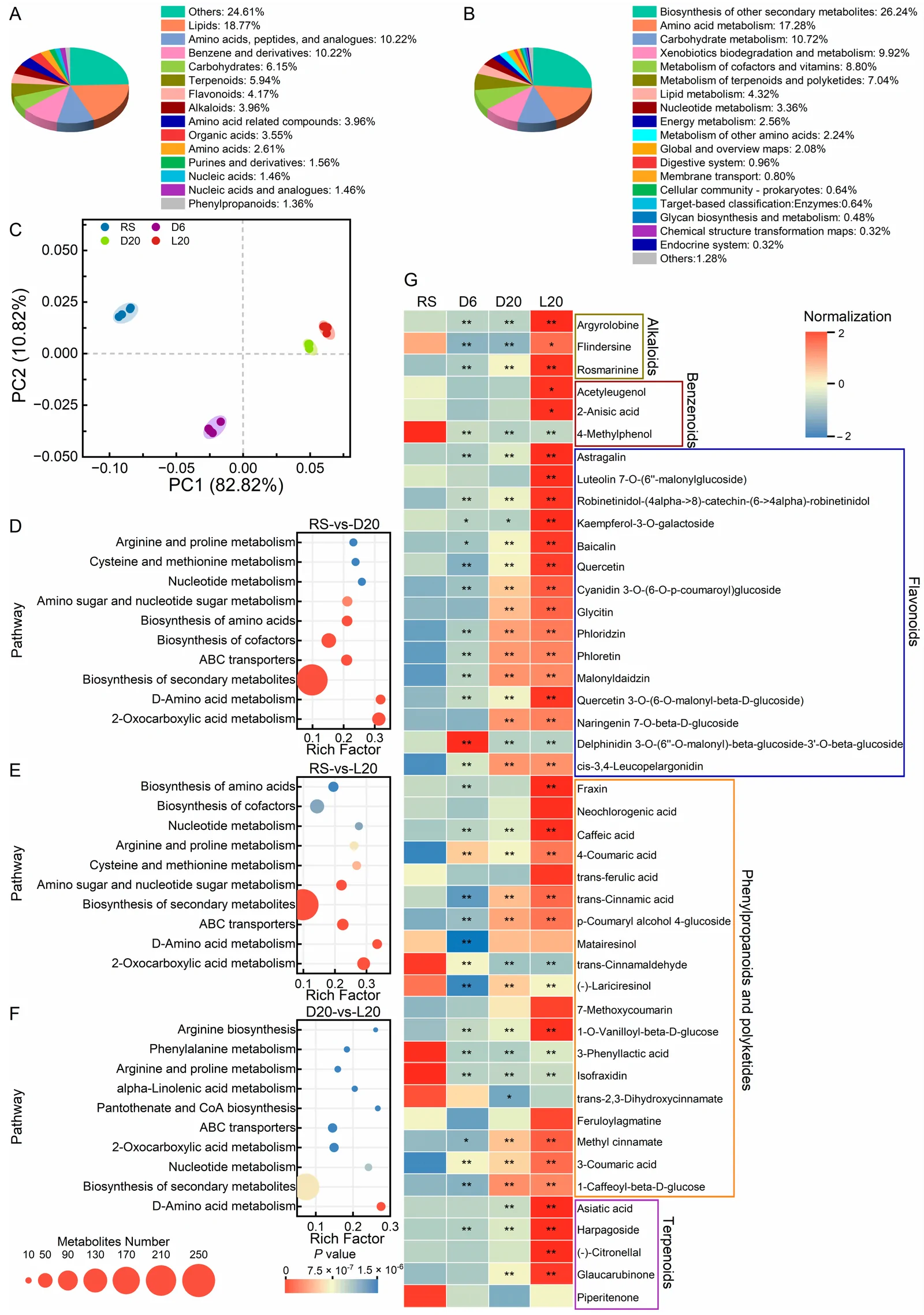
Phytochemical profiles identified by UHPLC-MS/MS during moringa seedling growth. (**A**) Classification of phytochemicals based on their chemical structures; (**B**) classification of phytochemicals based on KEGG pathway enrichment analysis; (**C**) principal component analysis (PCA) of phytochemicals detected at different sampling points. KEGG pathway enrichment analysis of significantly differentially accumulated phytochemicals between sampling points: (**D**) RS vs. D20; (**E**) RSs vs. L20; (**F**) D20 vs. L20. (**G**): Significantly differentially accumulated phytochemicals relative to RSs were selected for heatmap visualization (asterisks indicate significant differences compared with RSs). Statistical significance in the bubble charts and heatmap was assessed using the Wilcoxon test with Benjamini–Hochberg correction for multiple comparisons to obtain adjusted *p*-values (* *p* < 0.05, ** *p* < 0.01).

According to the UHPLC-MS/MS results, a total of 4059 phytochemical compounds were identified (Supplementary Table S3). Based on the classification of compounds by chemical type (Figure 4A), lipid-related compounds accounted for the largest percentage (18.77%), followed by amino acids (10.22%) and benzene-related compounds (10.22%), while phenylpropanoids accounted for the smallest percentage (1.36%). These compounds were subjected to KEGG pathway enrichment analysis (Figure 4B). Among all detected phytochemicals, the largest proportion was associated with the biosynthesis of other secondary metabolites (26.24%), followed by amino acid metabolism (17.28%) and carbohydrate metabolism (10.72%). Phytochemicals detected at different sampling points were subjected to principal component analysis (PCA) (Figure 4C), and the first two principal components (PC1 and PC2) explained 93.64% of the total variation. Differentially accumulated phytochemicals among different groups (RS vs. D20, RS vs. L20 and D20 vs. L20) were used for further KEGG pathway enrichment analysis (Figure 4D–F). Specifically, in all three comparisons, the pathway associated with D-amino acid metabolism exhibited the highest richness factors (0.32, 0.33 and 0.28, respectively), whereas the pathway associated with the biosynthesis of secondary metabolites contained the largest numbers of differential metabolites (221, 223 and 167, respectively). Differentially accumulated phytochemicals at different sampling points were further selected to generate a heatmap (Figure 4G). These phytochemicals were classified into five categories: alkaloids, benzenoids, flavonoids, phenylpropanoids and polyketides, and terpenoids. Additionally, the abundances of alkaloids (rosmarinine), flavonoids (astragalin, robinetinidol-(4alpha->8)-catechin-(6->4alpha)-robinetinidol, baicalin, cyanidin 3-O-(6-O-p-coumaroyl)glucoside, glycitin, phloretin, quercetin 3-O-(6-O-malonyl-beta-D-glucoside)), phenylpropanoids and polyketides (caffeic acid, 4-coumaric acid, 1-O-vanilloyl-beta-D-glucose, methyl cinnamate, 3-coumaric acid), and terpenoids (asiatic acid, harpagoside, glaucarubinone) were significantly higher after 20 days of growth compared with raw moringa seeds. On day 20, the abundances of these phytochemicals were also notably higher in the light-treated group than in the control group.

### 3.4. Phytochemicals Related to Functional Properties Revealed by Correlation Analyses

**Figure 5:**
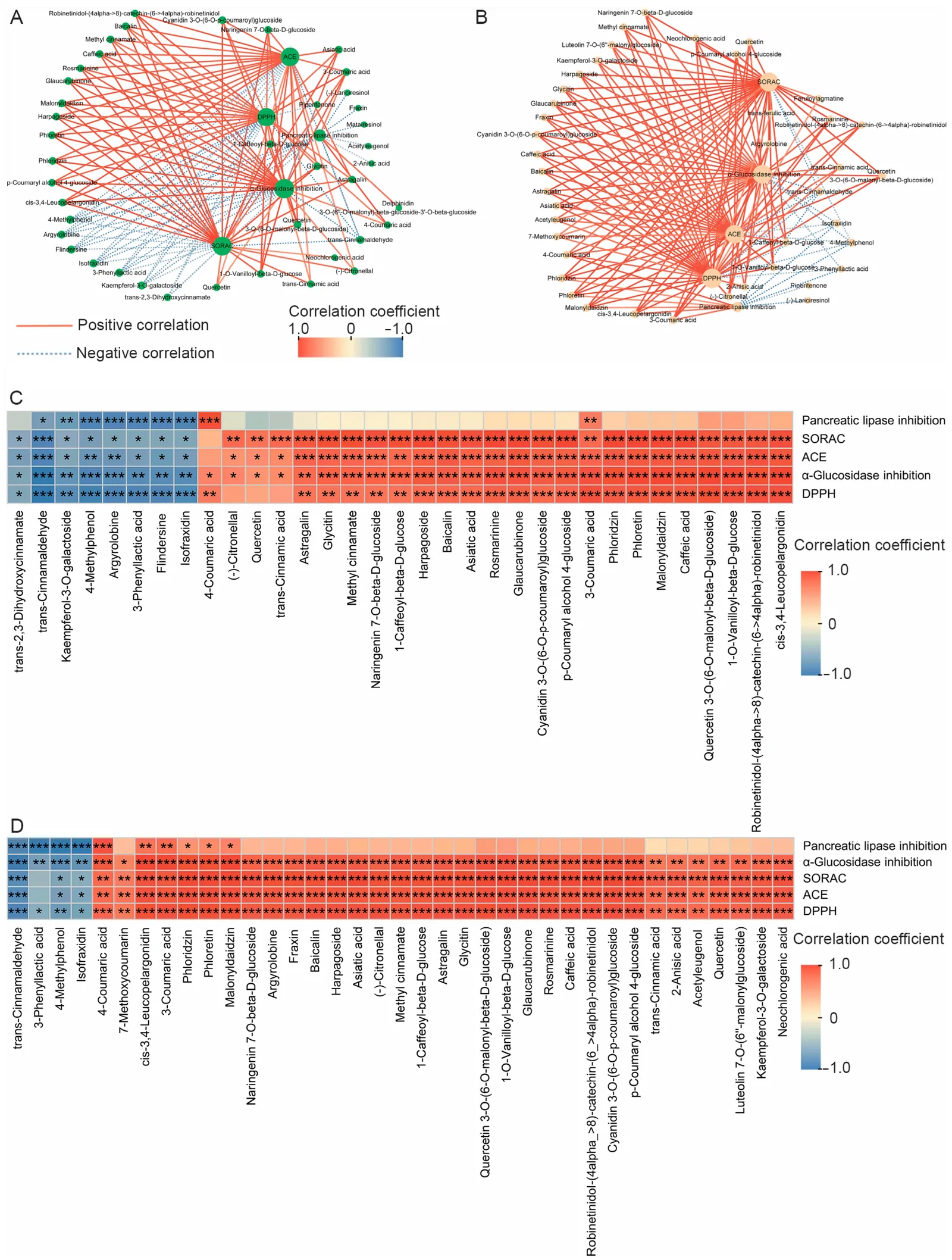
Correlation analyses between phytochemicals and functional properties of moringa seedlings. Molecular ecological network (MEN) profiles showing relationships between phytochemicals and functional properties in moringa seedlings cultivated under dark conditions (**A**) or light exposure (**B**). Positive and negative correlations are represented by red solid lines and blue dashed lines, respectively. Node size is proportional to the number of connections (degree) with other nodes in the network. Correlations between phytochemicals and functional properties are visualized using heatmaps for moringa seedlings cultivated under dark conditions (**C**) or light exposure (**D**). Spearman’s correlation coefficients were calculated to assess these relationships (* *p* < 0.05, ** *p* < 0.01, *** *p* < 0.001).

Phytochemicals that accumulated differentially in moringa seedlings after 20 days of growth compared with raw seeds were selected to construct correlation networks linking phytochemicals with functional properties (Figure 5A,B). For moringa seedlings grown in darkness, the hub node was α-glucosidase inhibition, with the highest node degree of 34. The hub phytochemicals included trans-cinnamaldehyde, 4-methylphenol, 3-coumaric acid, 3-phenyllactic acid, isofraxidin, kaempferol-3-O-galactoside, argyrolobine and flindersine, all of which showed a node degree of five. For moringa seedlings exposed to light, the hub node was DPPH scavenging capacity, with the highest node degree of 39. The hub phytochemicals included cis-3,4-leucopelargonidin, malonyldaidzin, 3-coumaric acid, 4-methylphenol, 4-coumaric acid, phloretin, trans-cinnamaldehyde and phloridzin, all of which showed a node degree of five. Nodes with a node degree greater than three were further selected for heatmap visualization (Figure 5C,D). For moringa seedlings grown in darkness, pancreatic lipase inhibition, α-glucosidase inhibition, DPPH scavenging capacity, SORAC and ACE inhibition showed a significant positive correlation with 4-coumaric acid (r^2^ = 0.97 ***), phloretin (r^2^ = 0.98 ***), quercetin 3-O-(6-O-malonyl-beta-D-glucoside) (r^2^ = 0.97 ***), phloridzin (r^2^ = 0.99 ***) and phloretin (r^2^ = 0.93 ***), respectively. For moringa seedlings exposed to light, pancreatic lipase inhibition, α-glucosidase inhibition, ACE inhibition, DPPH scavenging capacity and SORAC exhibited a strong positive correlation with 4-coumaric acid (r^2^ = 0.94 ***), 3-coumaric acid (r^2^ = 0.99 ***), phloridzin (r^2^ = 0.96 ***), phloretin (r^2^ = 0.98 ***) and 1-O-vanilloyl-beta-D-glucose (r^2^ = 0.99 ***), respectively.

### 3.5. Enzyme Activities and Gene Expressions in Phenylpropanoid Biosynthesis Pathway Increased During Moringa Seedling Growth

**Figure 6:**
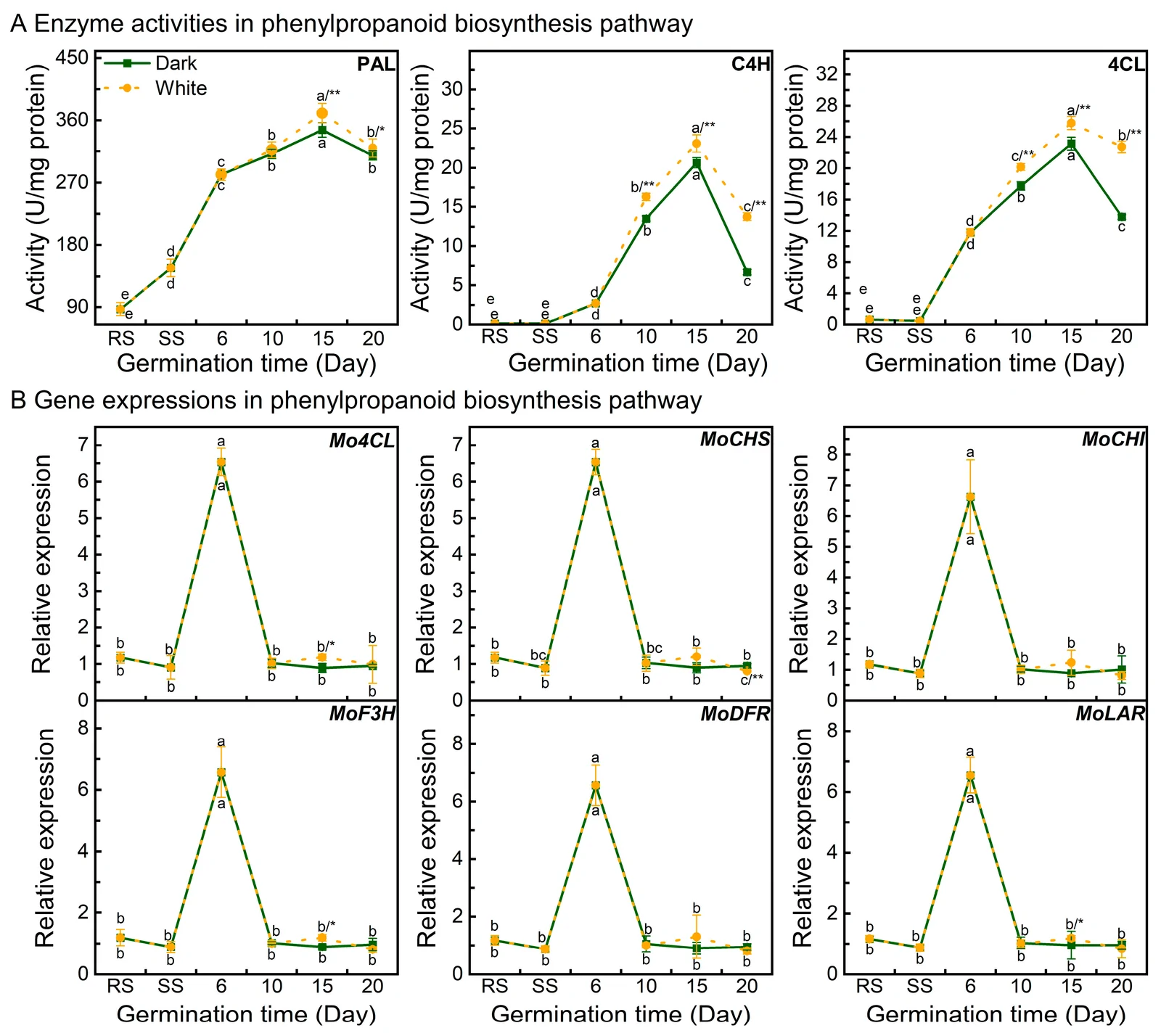
Changes in enzyme activities and gene expressions levels in phenylpropanoid biosynthesis pathway during growth of moringa seedling growth. (**A**) Activities of PAL, C4H, and 4CL; (**B**) relative expression levels of *Mo4CL*, *MoCHS*, *MoCHI*, *MoF3H*, *MoDFR*, and *MoLAR*). Statistical analyses were performed using an independent two-tailed *t*-test (two-group comparisons) and Duncan’s multiple range test (comparisons among multiple groups). Different lowercase letters indicate significant differences (*p* < 0.05) among sampling days within the same group, whereas asterisks indicate significant differences (* *p* < 0.05, ** *p* < 0.01) between the control and light-treated groups on the same sampling day.

The activities of all enzymes examined exhibited an increasing trend in activity during moringa seedling growth (Figure 6A), with the highest activities observed on day 15. Specifically, on day 15, the activities of PAL, C4H and 4CL reached 346.02, 20.66 and 23.15 U/mg protein in the control group, and 370.33, 23.10 and 25.79 U/mg protein in the light-treated group, respectively. From days 15 to 20, the activities of these enzymes in the light-treated group were significantly higher than those in the control. The expression levels of the genes involved in the phenylpropanoid biosynthesis pathway were further analyzed (Figure 6B). The expression levels of all the examined genes peaked on day 6 and then declined sharply. Compared with the control, the expression levels of *Mo4CL*, *MoF3H* and *MoLAR* in the light-treated group were noticeably higher on day 15.

## 4. Discussion

Under cultivation conditions with different light intensities, the group treated with 5 μmol/m^2^·s exhibited the highest growth parameters and respiratory intensity and the lowest electrolyte leakage. As light intensity increased beyond this level, growth parameters and respiratory intensity gradually decreased, whereas electrolyte leakage increased (Figure 2A–D). Under different photoperiod treatments, changes in growth parameters and respiratory intensity followed an inverted V-shaped pattern, in which these parameters reached their highest values at 8 h/d. From 0 to 8 h/d, these parameters increased gradually, whereas for photoperiods longer than 8 h/d, they decreased significantly with increasing light exposure duration (Figure 2E–G). However, electrolyte leakage followed a V-shaped pattern, with the lowest value observed at 8 h/d. Electrolyte leakage decreased from 0 to 8 h/d and increased markedly from 8 to 24 h/d (Figure 2H).

The efficiency of plant photosynthesis is closely related to light intensity and photoperiod [30]. Furthermore, Xiong et al. demonstrated that glucose produced from shoot photosynthesis in *Arabidopsis* could activate target-of-rapamycin (TOR) signaling by regulating glycolysis and mitochondrial bioenergetics to promote root meristem activation [31]. Consequently, as light intensity and photoperiod increased from their lowest levels (0 μmol/m^2^·s and 0 h/d) to their optimal levels (5 μmol/m^2^·s and 8 h/d), the increase in respiratory intensity might have resulted from an increase in glucose supply through photosynthesis and greater growth demands, which promoted glucose oxidation to generate ATP for cell division and expansion, as reflected by increased hypocotyl length and biomass accumulation. Electrolyte leakage is widely used as an indicator of membrane stability and oxidative stress in plant tissues, since electrolyte leakage is often mediated by reactive oxygen species (ROS)-activated plasma-membrane ion channels accompanying K^+^ efflux, metabolic reprogramming and programmed cell death [32]. In this study, moringa seedlings cultivated under light intensities and photoperiods below the optimal levels exhibited higher electrolyte leakage, possibly because insufficient photon capture limited net carbon gain and productive respiration, thereby restricting the ATP and antioxidant supply required for cell membrane repair and increasing susceptibility to oxidative damage. A comparable response was observed in *Carpinus betulus* L. seedlings, in which reductions in light intensity were accompanied by marked increases in malonaldehyde content and relative electrolytic conductivity [33]. In contrast, under optimal light conditions (intensity and photoperiod), ROS may still be generated and function primarily as signaling molecules that activate antioxidant defenses, thereby maintaining oxidant–antioxidant homeostasis rather than causing membrane damage. When light intensity and photoperiod exceeded their optimal levels, photoinhibition and photo-oxidation could occur because plants were exposed to light conditions exceeding the capacity of their photosynthetic apparatus to utilize the absorbed energy [34]. Previous research has shown that increased light radiation intensity could inhibit the activity of the pyruvate dehydrogenase complex, limiting the conversion of pyruvate to acetyl-CoA. Consequently, CO_2_ release from the tricarboxylic acid (TCA) cycle could decrease, leading to reduced respiratory intensity [35]. Furthermore, continuous light exposure could inhibit respiratory intensity through two mechanisms: decreased activity of ribulose-1,5-bisphosphate carboxylase/oxygenase, which may reduce the supply of precursors to glycolysis and the TCA cycle, and structural changes in leaves, such as increased thickness or density [36]. In the present study, exposure of moringa seedlings to light intensities or photoperiods exceeding the optimal levels inhibited respiratory intensity, potentially reducing ATP production and contributing to growth inhibition and lower biomass accumulation. Furthermore, the excess excitation energy resulting from light exposure could promote ROS production (particularly singlet oxygen, ^1^O_2_), contributing to photo-oxidative damage, membrane disruption, chloroplast ultrastructural damage, and plant cell death [37]. Thus, the increased electrolyte leakage observed under light intensities and photoperiods exceeding the optimal levels might be attributed to excessive ROS generation through photo-oxidation, resulting in lipid peroxidation and cell membrane damage. Antioxidant capacity was evaluated using DPPH scavenging capacity and SORAC, the differences in which were used to estimate the ability to quench artificial free radical (DPPH) or superoxide anion free radicals (SORAC). In this study, DPPH scavenging capacity showed significant positive correlations with quercetin 3-O-(6-O-malonyl-beta-D-glucoside) in the control group and phloretin in the light-treated group. Furthermore, SORAC showed the strongest positive correlations with phloridzin in the control group and 1-O-vanilloyl-beta-D-glucose in the light-treated group (Figure 5C,D). The antioxidant activity of moringa seedlings might be attributed, at least in part, to these phytochemicals, whose antioxidant properties have been demonstrated in previous studies [38,39,40]. Except for phloretin, the compounds mentioned above are phenolic or flavonoid glycosides containing quercetin, phloretin, or vanillin-related structural components. The radical scavenging effects of these compounds may be associated with hydrogen atom donation from phenolic groups and electron transfer, whereas glycosylation may influence their solubility and bioavailability. The pancreatic lipase inhibition rate showed a notable positive correlation with 4-coumaric acid in both the control and light-treated groups (Figure 5C,D). 4-Coumaric acid is a hydroxycinnamic acid that has been reported to inhibit pancreatic lipase through interactions with its active site, with the resulting complex stabilized by hydrogen bonding and hydrophobic interactions, including π-stacking [41]. Consequently, the pancreatic lipase inhibitory activity of moringa seedlings could be partly associated with the accumulation of 4-coumaric acid. The α-glucosidase inhibition rate showed notable positive correlations with phloretin in the control group and 3-coumaric acid in the light-treated group (Figure 5C,D). The α-glucosidase inhibitory activities of phloretin and 3-coumaric acid have been reported previously, and the underlying mechanisms may involve interactions with the enzyme’s active site through hydrophobic interactions, van der Waals forces, and hydrogen bonding [42,43]. Thus, the α-glucosidase inhibitory activity of moringa seedlings might be partly attributed to phloretin and 3-coumaric acid. The ACE inhibition rate showed notable positive correlations with phloretin in the control group and phloridzin in the light-treated group (Figure 5C,D). Previous studies have demonstrated the ACE inhibitory activities of these compounds, potentially through the inhibition of ACE catalytic activity [44,45]. Therefore, the ACE inhibitory activity of moringa seedlings might be associated with the accumulation of these two phytochemicals. One notable finding was the accumulation of total phenolics, total flavonoids and phytochemicals associated with functional properties (quercetin 3-O-(6-O-malonyl-beta-D-glucoside), phloretin, phloridzin, 1-O-vanilloyl-beta-D-glucose, 4-coumaric acid and 3-coumaric acid) during moringa seedling growth (Figure 3 and Figure 4G). Because these phenolic and flavonoid compounds are associated with the phenylpropanoid biosynthesis pathway, the subsequent analyses showed that the enzyme activities in this pathway increased and reached their highest levels on day 15 (Figure 6A), whereas the expression levels of the corresponding genes peaked on day 6 (Figure 6B). This difference might be attributed to a time lag between gene transcription and the accumulation of enzymatic activity. Because the genes encoding PAL and C4H in moringa were not available in the NCBI database, their expression levels were not determined. The expression levels of genes involved in phenolic and flavonoid biosynthesis showed the greatest increases between the soaked-seed stage (SSs) and day 6 of seedling growth. During the same period, PAL and 4CL activities also exhibited their largest increases, suggesting that phytochemical accumulation during moringa seedling growth is associated with the regulation of phenylpropanoid biosynthesis-related genes and enzymes. Dry seeds are metabolically quiescent, but after soaking, early germination is the high-risk phase, as ROS are produced through respiration, lipid β-oxidation, reserve mobilization and cell-wall loosening. Under these conditions, the expression of genes involved in the phenylpropanoid pathway may increase, promoting phenolic and flavonoid accumulation to counteract excessive ROS and maintain cellular stability during early seedling emergence. Similarly, previous studies have reported widespread transcriptional activation during Arabidopsis seed germination and antioxidant accumulation during moringa seed germination [46,47]. However, once antioxidant defenses are established and flavonoids have accumulated, the subsequent decline in phenylpropanoid pathway gene expression after day 6 may reflect a reduced requirement for early protective responses. At this stage, resource allocation may shift from protective secondary metabolite production toward seedling growth, including root elongation, shoot expansion, photosynthetic apparatus development, and cell-wall formation. Another notable finding was that the contents of total phenolics, total flavonoids and phytochemicals associated with functional properties (quercetin 3-O-(6-O-malonyl-beta-D-glucoside), phloretin, 1-O-vanilloyl-beta-D-glucose, 4-coumaric acid and 3-coumaric acid) were significantly higher in the light-radiated group compared with the control (Figure 3 and Figure 4G). Similar results have been reported in *Moringa oleifera* callus cultures, where the accumulation of total phenolics, total flavonoids, and phytochemicals (kaempferol, neochlorogenic acid, quercetin, luteolin, apigenin, and p-coumaric acid) was significantly enhanced by white light exposure [48]. Further analyses revealed that PAL, C4H, and 4CL activities were markedly higher in the light-treated group than in the control from days 15 to 20 (Figure 6A). The expression levels of *Mo4CL*, *MoF3H*, and *MoLAR* were also notably higher in the light-treated group on day 15 (Figure 6B). Liu et al. reported that light-activated photoreceptors, including phytochromes, cryptochromes, and UV Resistance Locus 8 (UVR8), could suppress the activity of the Constitutive Photomorphogenic 1 (COP1)/Suppressor of Phytochrome A-105 (SPA) complex and De-Etiolated 1 (DET1), thereby promoting the stabilization of Elongated Hypocotyl 5 (HY5). HY5 may subsequently act as a transcription factor to activate regulatory genes (MYBs) and structural genes (PAL, CHS, F3H, DFR, and LDOX) involved in the phenylpropanoid pathway [49]. In this study, broad-spectrum white light, particularly its blue and red wavelengths, may have activated cryptochrome- and phytochrome-dependent signaling, suppressed COP1/SPA-mediated repression, stabilized HY5, and activated MYB/HY5-regulated phenylpropanoid genes (*MoPAL*, *MoC4H*, *Mo4CL*, *MoF3H*, and *MoLAR*). These processes may have enhanced PAL, C4H, and 4CL activities and promoted metabolic flux toward p-coumaric acid, flavonoids, and other protective phenolic compounds. Consequently, the C4H and 4CL activities were notably higher in the light-treated group than in the control on day 20 (Figure 6A).

In this study, the white LED light intensity and photoperiod were optimal at (5 μmol/m^2^·s and 8 h/d) based on the growth and physiological parameters of the moringa seedlings during cultivation. Subsequently, phytochemical accumulation and its potential underlying mechanisms were investigated under these optimal lighting conditions. However, in addition to light intensity and photoperiod, spectral composition is another important factor influencing the growth and phytochemical accumulation of edible microgreens. According to the review by Borrelli et al. [50], blue (B) and red (R) light combinations at ratios of B50:R50 or B30:R70 were among the most effective for promoting microgreen growth and nutrient accumulation. Specifically, a B50:R50 light spectrum markedly promoted mineral nutrient accumulation and fresh biomass production in kale seedlings [51]. Similarly, growth, fresh weight accumulation, antioxidant capacity, and total soluble solid content were markedly enhanced in microgreens, including mustard, lettuce, radish, and broccoli, under a B30:R70 light combination [52]. Moreover, compared with visible light alone, the combination of visible light and UV-A significantly enhanced growth and antioxidant contents in microgreens, including basil, beet, and pak choi [53]. In this study, the effects of light intensity and photoperiod on moringa seedling growth and phytochemical accumulation were investigated under a fixed light spectrum. Future research should investigate the effects of monochromatic light and different wavelength combinations to more precisely regulate the growth and quality of moringa seedlings.

## 5. Conclusions

Overall, the optimal white LED light conditions (light intensity and photoperiod) for moringa seedling cultivation were identified, which promoted fresh weight accumulation and hypocotyl elongation by upregulating respiratory intensity and reduced electrolyte leakage. During seedling growth, total phenolics, total flavonoids, and other phytochemicals accumulated, and functional properties were enhanced, as the expression of genes (*Mo4CL*, *MoF3H*, *MoLAR*) and activities of enzymes (PAL, C4H, 4CL) in the phenylpropanoid pathway were upregulated by light radiation. Consequently, white LED light treatment at an intensity of 5 μmol/m^2^·s and a photoperiod of 8 h/d may serve as an effective approach for promoting moringa seedling growth and phytochemical accumulation, supporting their potential application as functional microgreens.

## Data Availability

The original data obtained in this study are included in the article/Supplementary Material. Further inquiries can be directed to the corresponding authors.

## Supplementary Materials

The following supporting information can be downloaded at: https://www.mdpi.com/article/10.3390/foods15213960/s1. The data supporting this article are included in the Supplementary Material under the file name Raw Data. Other Supplementary Materials include the spectral energy distribution of the white LED lamp (Supplementary Figure S1); morphological changes in moringa seedlings cultivated under different light radiation intensities (Supplementary Figure S2) and different light radiation periods (Supplementary Figure S3); changes in hypocotyl diameter of moringa seedlings exposed to different light intensities during growth (Supplementary Table S1) and different light periods during growth (Supplementary Table S2); phytochemicals detected by UHPLC-MS/MS during moringa seedling growth (Supplementary Table S3); primers used for quantitative real-time PCR (Supplementary Table S4) [54,55,56].
