# Supplementary material for: White LED Light Radiation Promotes Growth and Phytochemical Accumulation in Moringa Seedlings as Promising Functional Microgreen
Source: Foods. 2026 Nov 4;15(21):3960. doi: 10.3390/foods15213960 (PMC13762651; doi:10.3390/foods15213960)
Supplement: Supplementary file 1 [file foods-15-03960-s001.zip › Supplementary Figure S1-S3.pdf]

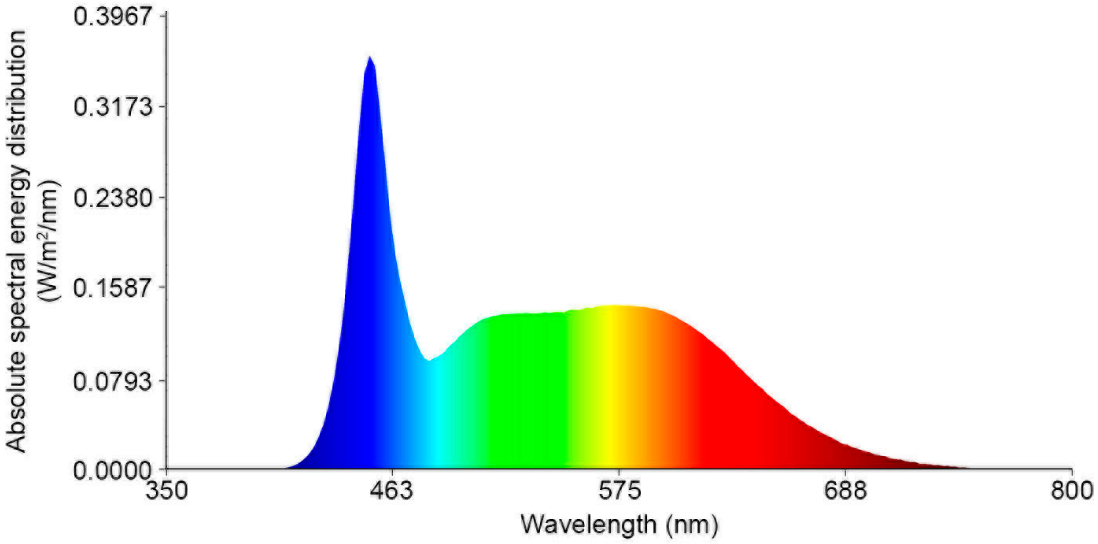

Supplementary Figure S1. Spectral energy distribution of white LED lamp

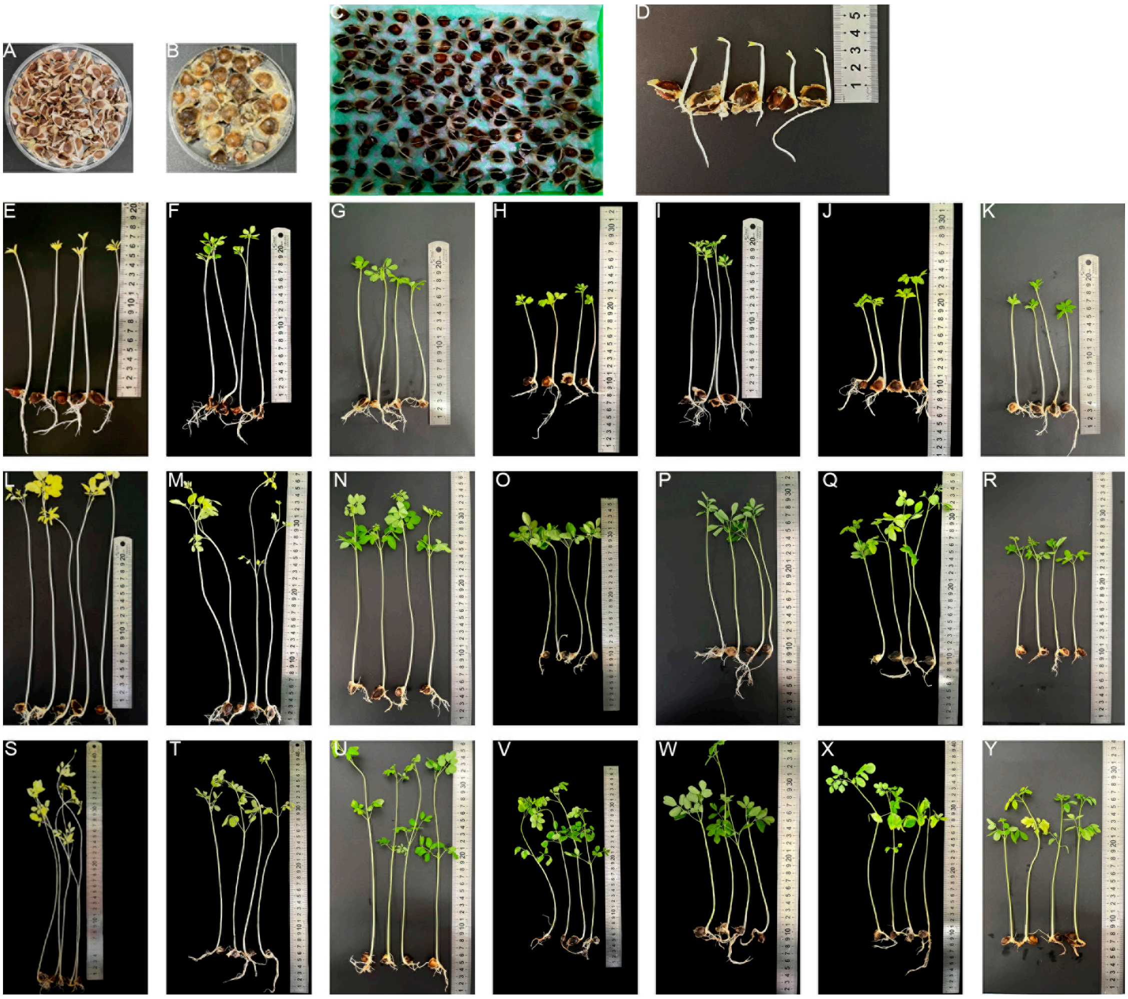

Supplementary Figure S2. Morphological changes in moringa seedlings cultivated under different light intensities during growth. (A): Raw seeds; (B): seeds after 24 h of soaking; (C): seeds after 5 days of pregermination; (D): sprouts after 6 days of germination; (E–K): morphological changes in moringa seedlings cultivated under different light intensities on day 10; (L–R): morphological changes in moringa seedlings cultivated under different light intensities on day 15; (S–Y): morphological changes in moringa seedlings cultivated under different light intensities on day 20. The light intensities applied to the different treatment groups were 0, 5, 15, 30, 60, 120 and 360 μmol/m².s, respectively.

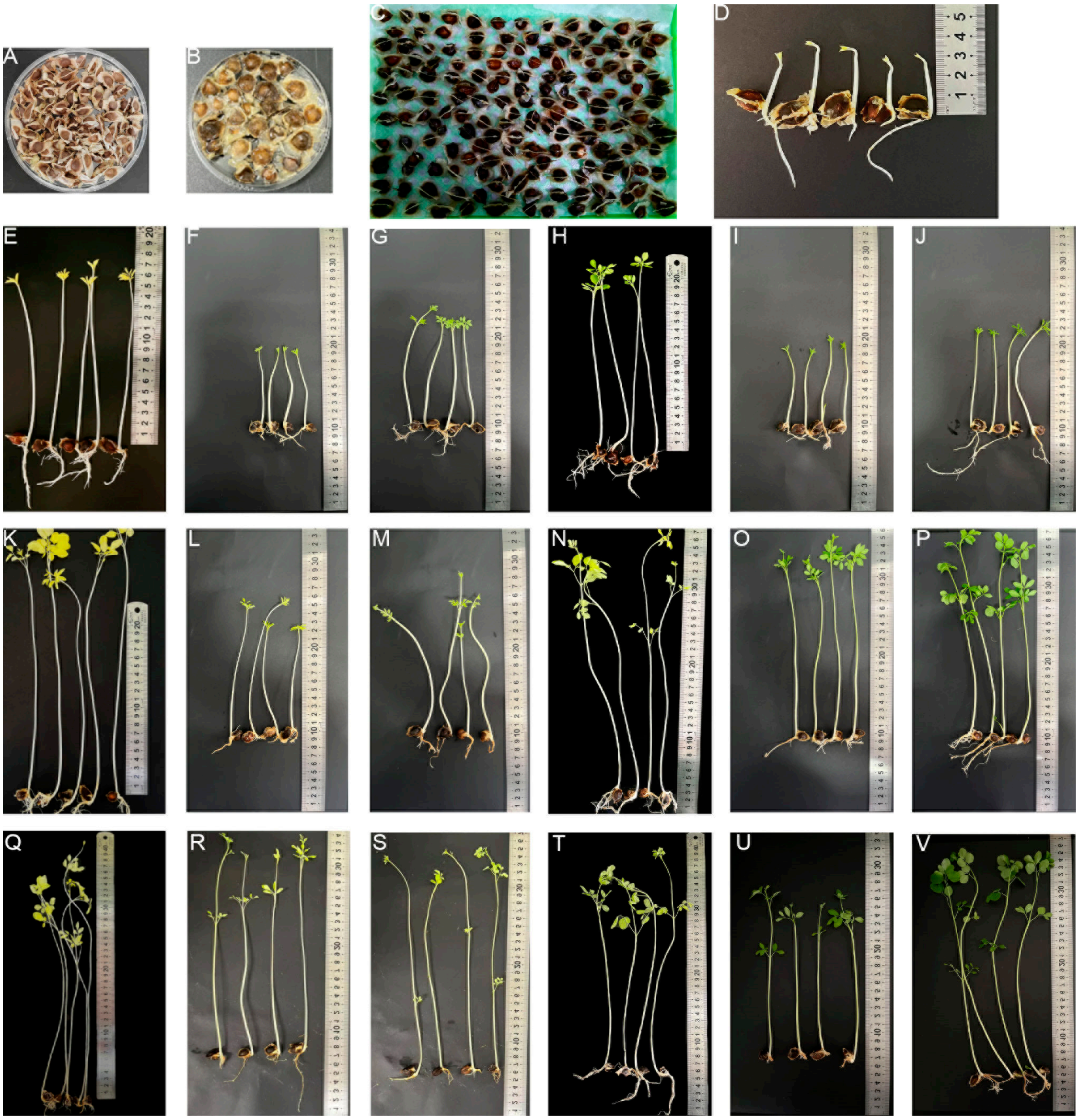

Supplementary Figure S3. Morphological changes in moringa seedlings cultivated under different photoperiods during growth. (A): Raw seeds; (B): seeds after 24 h of soaking; (C): seeds after 5 days of pregermination; (D): sprouts after 6 days of germination; (E–J): morphological changes in moringa seedlings cultivated under different photoperiods on day 10; (K–P): morphological changes in moringa seedlings cultivated under different photoperiods on day 15; (Q–V): morphological changes in moringa seedlings cultivated under different photoperiods on day 20. The photoperiods applied to the different treatment

groups were 0, 2, 4, 8, 12 and 24 h/d, respectively.
